# Stretchable Binary Fresnel Lens for Focus Tuning

**DOI:** 10.1038/srep25348

**Published:** 2016-05-03

**Authors:** Xueming Li, Lei Wei, René H. Poelma, Sten Vollebregt, Jia Wei, Hendrik Paul Urbach, Pasqualina M. Sarro, Guo Qi Zhang

**Affiliations:** 1 Department of Microelectronics, Delft University of Technology, Delft, 2628 CT, The Netherlands; 2 Department of Imaging Physics, Delft University of Technology, Delft, 2628 CH, The Netherlands

## Abstract

This paper presents a tuneable binary amplitude Fresnel lens produced by wafer-level microfabrication. The Fresnel lens is fabricated by encapsulating lithographically defined vertically aligned carbon nanotube (CNT) bundles inside a polydimethyl-siloxane (PDMS) layer. The composite lens material combines the excellent optical absorption properties of the CNT with the transparency and stretchability of the PDMS. By stretching the elastomeric composite in radial direction, the lens focal length is tuned. Good focusing response is demonstrated and a large focus change (≥24%) was achieved by stretching lenses up to 11.4%.

A tuneable lens plays an important role in adaptive illumination, beam shaping and optical communication, where controllable focal length is required for many scenes^1^. Various and remarkable techniques to obtain focus change are proposed in the literature. For example, a lens based on liquid filled elastomer shells was used to create a dual mode meniscus lens, by tuning the pressure of the micro chamber^2^. However, it has the drawback of shape instability and temperature sensitivity. Less robust solutions have also been proposed which rely on electrostatically controlling the contact angle of a liquid lens on a substrate^3^,^4^. In another approach, a fixed optical element was mounted on a large displacement actuator to adjust the lens focal length^5^,^6^. However, this results in a bulky system not suitable for miniaturization due to the assembly of individual parts. Depending on the applications, the reported concepts have different focal length ranges. However, significant challenges are encountered when the systems are miniaturized and when the size and shape of the lens needs to be adaptive.

A micro binary amplitude Fresnel lens uses micro-structured patterns to spatially modulate the intensity distribution of the light passing through. The micro Fresnel lens has been demonstrated in applications such as holograms^7^, 3D integral imaging^8^, and laser processing^9^. When it comes to the miniaturization of lenses, traditional optics suffer from lens surface curvature, small numerical apertures and increased difficulty in fabrication. However, diffractive optics have the advantages of being flat and thin. Furthermore, their structure shows great compatibility with micro fabrication technologies, thus bringing more freedom to the lens design^10^. In diffractive optics, the opaque region is critical for the redistribution of light. Materials such as aluminium^11^, silicon^12^, carbon nanotubes (CNT)^13^ and graphene^14^ have been reported as suitable opaque materials. Among them, CNT is an excellent candidate material due to the very high optical absorption of the material^15^. Furthermore, the fast growing CNT bundles in photo-lithographically defined patterns, have been recognized as an excellent structural material for the fabrication of high-aspect-ratio deformable 3D micro-structures^16^. Most of the reported micro Fresnel lenses are fabricated using rigid materials and cannot be adaptive. However some recent progress has been made using silicon nanowires combined with a flexible polymer to show that a large field of view change is possible by bending the substrate^17^.

In this paper, we report on a micro Fresnel lens composed of CNT embedded inside polydimethylsiloxane (PDMS), a polymer with great optical transparency, stretchability and bio-compatibility^18^. The PDMS is employed as both the transparent optical lens material and as stretchable substrate. The CNT, with excellent light absorption properties, is employed as the opaque material for the binary amplitude Fresnel lens^19^. The lens formed by the CNT/PDMS composite changes its focal length by stretching the substrate.

## Results

### Design of the tuneable Fresnel lens

**Figure 1:**
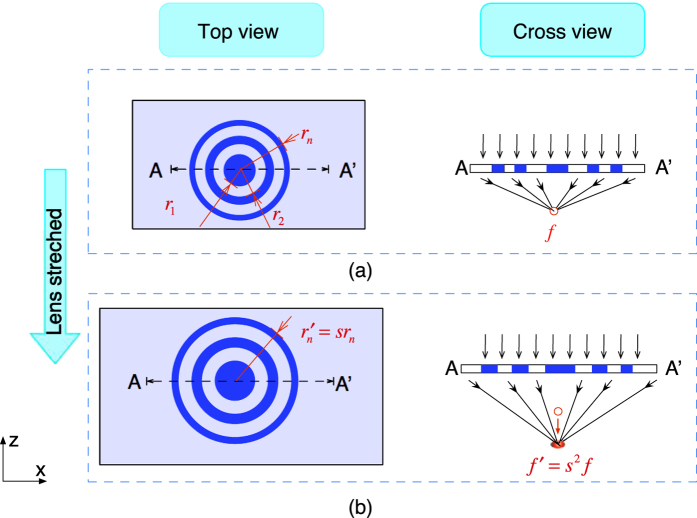
Schematic illustration of the tuneable Fresnel lens. When stretching the flexible substrate radially by *s* from (**a**) to (**b**), the radius of the *n*_*th*_ zone increases from *r*_*n*_ to 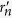 and the focal length changes from *f* to *f*′ by a factor of by *s*^2^.

A binary amplitude Fresnel lens consists of alternating opaque and transparent zones, see Fig. 1a. The focal length *f* of the lens is related to the zone number n (n = 1, 2, ...), the zone radius *r*_*n*_ and the wavelength *λ* of the illuminating light^20^,


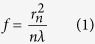


When the zone radius *r*_*n*_ increases by the stretching factor *s* to 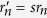, the new focal length *f*′ of the lens increases by *s*^2^,


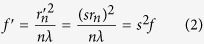


Therefore, we can design a lens with adaptive size and highly sensitive focus tuning, as schematically depicted in Fig. 1.

The Fresnel lens configuration presented here utilizes CNT as the opaque areas, and PDMS as transparent material as well as stretchable substrate. A lens with focal length of 7 *mm* is designed. According to Equation 1, the diffractive lens pattern has an innermost radius *r*_1_ of 66.7 *μm*, with 30 zones, as it provides good focusing performance with a reliable fabrication process. Higher zone numbers will provide sharper focus by suppressing higher order focus. However, it also requires smaller pitches for the high order zones, which may bring challenges to the CNT growth process. The CNT are 10 *μm* high as this height promises perfect optical absorption^15^. The PDMS layer has a thickness of 2 *mm*, making it easy to handle, while keeping great stretchability of the lens.

**Figure 2:**
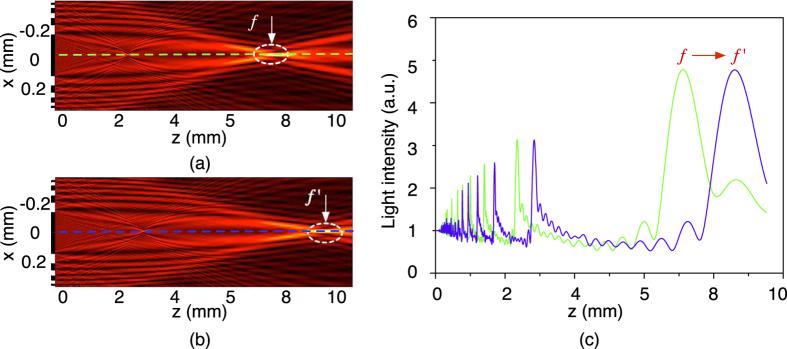
Computed wave propagation and focus change when the lens size is changed. (**a**) Light focuses at 7 *mm* after it propagates through the lens; (**b**) When the lens is radially stretched by 10%, light redistributes and focuses at around 8.47 *mm*; (**c**) The light intensity along the z-axis of the focal plane, shows a clear shift of the peak intensity, indicating a focal point change from *f* to *f*′.

To analyse the wave propagation and focusing performance of the tuneable Fresnel lens, a 2D computation of the distribution after light propagates through the lens was made with Matlab R2015a (Mathworks, B.V.). Figure 2 compares the lens, designed for *λ* = 635 *nm*, before and after stretching by 10%.

Figure 2a shows the calculated focal point distance of the lens as indicated by the bright area outlined by the white ellipse. This indicates that the computed focal length is about 7 *mm*, which closely matches our initial design. Figure 2b shows the light redistribution and focus point at around 8.47 *mm* when the size of the Fresnel lens pattern is increased by 10%. Figure 2c depicts the light intensity along the z-axis for the configuration in Fig. 2a,b. The light intensity reaches a peak at z = 7 *mm* and 8.47 *mm* separately, indicating a focus change of about 1.47 *mm* (Δ*r* = *s*^2^*r*) is realized when the size of the lens increased by 10% (*s* = 1.1). This demonstrates the exponential increase in focal distance.

### Fabrication of the Fresnel lens

**Figure 3:**
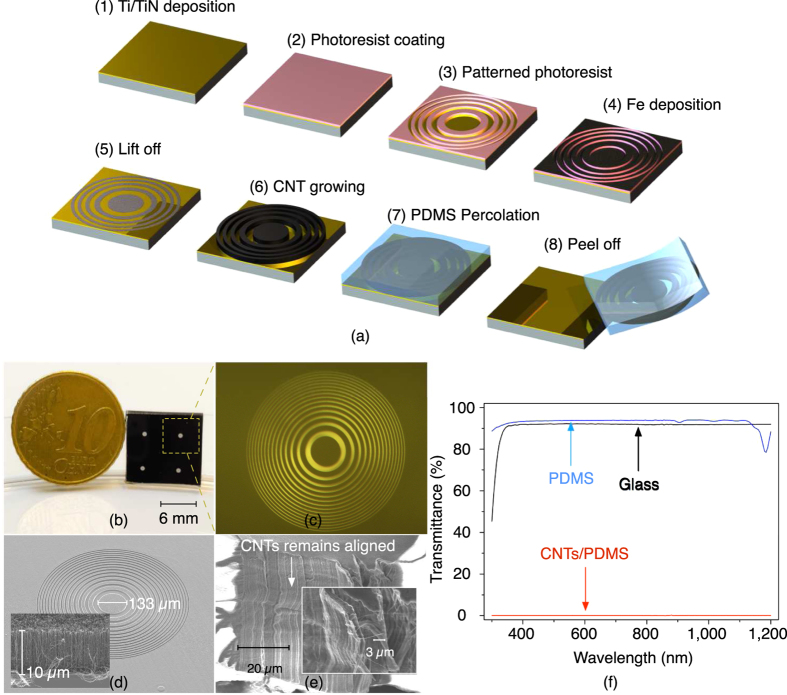
(**a**) Schematic illustration of the microfabrication process of the stretchable Fresnel lens (not to scale); (**b**) Fabricated device containing 2 × 2 lens units (*f* = 7 *mm*), with each unit size 6 × 6 *mm*^2^; (**c**) Optical microscope image of one lens; (**d**) Tilted SEM image of the diffractive CNT pattern, the inset shows a close up view of the vertically aligned CNT which are 10 *μm* in height (before PDMS percolation); (**e**) SEM image of the CNT with the PDMS percolated thoroughly into the CNT bundles; (**f**) The measured transmittance of the transparent PDMS area and the opaque CNT/PDMS area, with glass as reference. The PDMS has a transmittance of 93.9%, higher than glass (91.8%), while the CNT/PDMS has transmittance of 0.06%.

The main fabrication steps are schematically depicted in Fig. 3a: (1) Ti/TiN (10 *nm*/50 *nm*) was sputtered on a silicon wafer to prevent diffusion of the catalyst into the substrate; (2) 1.4 *μm* photoresist was coated and (3) patterned on the wafer; (4) Iron (5 *nm*) was then evaporated as catalyst, followed by (5) a lift-off process to define the CNT growth regions; (6) Vertically aligned CNT bundles (10 *μm* in height) were grown in an AIXTRON Black Magic chemical vapour deposition reactor, at a temperature of 550 °C in 10 min using conditions as specified in ref. 21; (7) PDMS was then mixed with the curing agent by a ratio of 10:1 and poured on the horizontal silicon wafer substrate with the defined CNT patterns. Step (7) is then followed by degassing at room temperature for 30 min, and curing at 65 °C for 1 hour. Degassing before curing is important as to prevent trapping of gas bubbles inside the PDMS; (8) The device is released by peeling off the PDMS layer together with the encapsulated CNT from the silicon substrate.

A fabricated device containing 2 × 2 lens units, with each unit 6 × 6 *mm*^2^ in size, is shown in Fig. 3b. An optical microscopy image of a single lens is shown in Fig. 3c. A scanning electron microscopy (SEM) image (tilted view) of a single lens unit, with 7 *mm* in focal length and an innermost zone diameter of 133 *μm*, before PDMS infiltration, is shown in Fig. 3d. The inset shows a close up view of the vertically aligned CNT and the well aligned and dense CNT forest structure. Figure 3e shows a SEM image of the CNT/PDMS composite which is used to inspect the penetration of the polymer into the CNT forest. We observe that the CNT inside the PDMS are still well aligned after the PDMS percolation, thus maintaining the CNT patterns and the excellent optical absorption properties. Furthermore, the thorough penetration of PDMS promises good flexibility for the composite, which is important for the tuneability of the lens. The transparency of the PDMS material and the absorption of the opaque area of the patterned CNT are fundamental to the optical performance of the Fresnel lens. The transmittance of the transparent PDMS area and the opaque CNT/PDMS area are measured with a spectrophotometer (PerkinElmer 950), as shown in Fig. 3f. The PDMS area has a transmittance of around 93.9% in the visible light spectrum, which is higher than glass (91.8%); while the CNT/PDMS area shows a transmittance of 0.06% over the 300–1,200 *nm* spectral range. The great transparency of the PDMS area and the low transmittance of the CNT/PDMS composite area provide great contrast between the transparent areas and opaque areas, which in turn promise the Fresnel lens good focusing performance.

**Figure 4:**
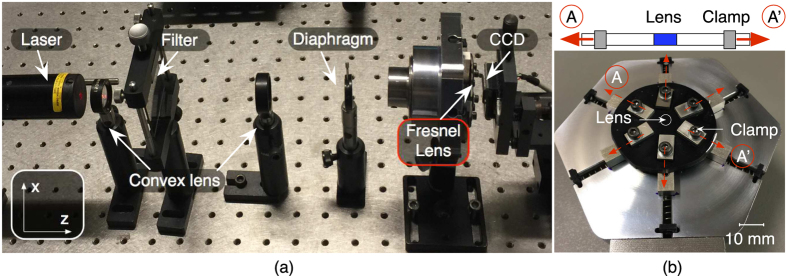
(**a**) The experimental setup used for the lens characterization. A 635 *nm* laser is used to illuminate the Fresnel lens, which is adjusted by two convex lenses, a filter and a diaphragm to improve collimation. A CCD camera captures the image of the focal region of the lens. By moving the camera along the z-axis, the focus intensity distribution can be detected; (**b**) The lens is maintained on a customized sample holder with six clamps for holding and stretching the lens in the radial direction.

The measurement setup used to characterize the stretchable Fresnel lens is shown in Fig. 4a. The lens is clamped on a customized holder and is illuminated by a 635 *nm* laser, which is adjusted by two convex lenses, a filter and a diaphragm to improve the collimation. Diffraction patterns are captured by a charge-coupled device (CCD) camera as the light propagates through the lens. The holder used to stretch the lens in radial direction is shown in Fig. 4b. Six symmetrically distributed clamps are maintaining the lens on the holder. Each clamp can be adjusted by the same amount in increments of 0.289 *mm* in radial direction.

### Optical performance of the lens

**Figure 5:**
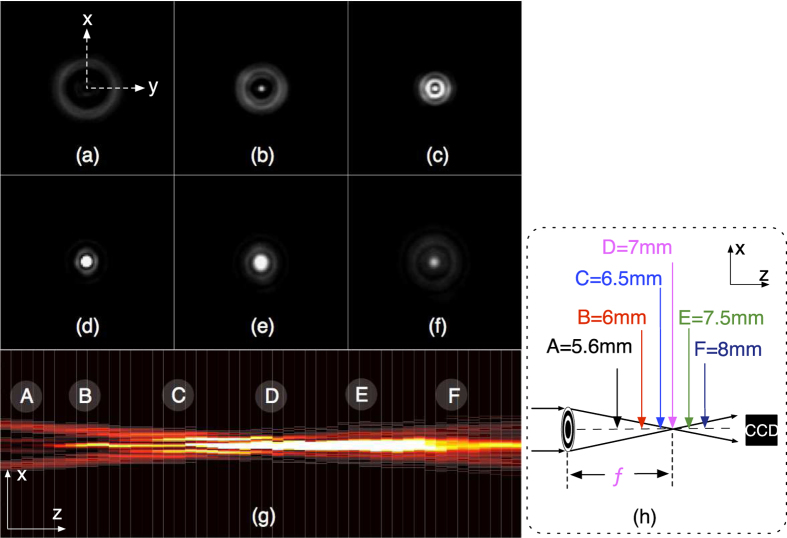
Diffraction patterns along the z-axis near the focal plane captured by a CCD camera. (**a**) A ring is observed at position A; (**b**) Diffraction pattern size becomes smaller and a bright spot appears in the centre at position B; (**c**) The light spot becomes smaller and brighter at C; (**d**) An clear light spot is formed at D, which indicates the focal plane; (**e,f**) moving further towards position E and F the pattern becomes dimmer, wider and it eventually defocuses; (**g**) The reconstructed intensity distribution from z = 5.6 *mm* to 8.05 *mm* are shown; The captured pictures from (**a–f**) are indicated as the positions A-F as shown in (**h**). The intensity distribution can be compared with the computed intensity reported in Fig. 2a in the regime of z = 5.6 *mm* to 8.05 *mm*.

Light distribution of the lens is measured near the focal plane (z = 7 *mm*). A CCD camera is employed to capture the diffraction images along the z-axis. With the collected images an intensity distribution is reconstructed. The captured pictures as shown in Fig. 5a–f, are indicated as the positions A-F (with z = 5.6 *mm*, 6 *mm*, 6.5 *mm*, 7 *mm*, 7.5 *mm*, 8 *mm* separately) in Fig. 5h. Figure 5a shows that at position A, a ring is obtained. The diffraction pattern at position B becomes smaller and brighter, and a light spot started to form in the centre (Fig. 5b). The light spot becomes more bright and smaller when moving to position C (Fig. 5c). A focused light spot is then observed at position D (Fig. 5d), which indicates the position the focal plane. Beyond the focal plane, the light spot becomes dimmer and wider. Defocused patterns are observed at E (Fig. 5e) and F (Fig. 5f), with the light spot getting smaller. The overview of the intensity distribution from z = 5.6 *mm* to z = 8.05 *mm* is shown with a reconstructed intensity map, by collecting 50 distributed images of the diffraction patterns along the z-axis, with an increment of 0.05 *mm*. The diffraction patterns are normalized (white represents the maximum light intensity and black is the off state of the camera). The intensity profiles from the x-axis (y = 0) of each image were then extracted and reconstructed to an intensity map, as shown in Fig. 5g. The intensity along z-axis starts with a low intensity in the centre at A, and forms a light spot at around B and C, after it reaches a peak at D, which also indicates the focal point, it then becomes wider at around E and defocused at around F. The intensity distribution can be compared with the computed intensity reported in Fig. 2a.

**Figure 6:**
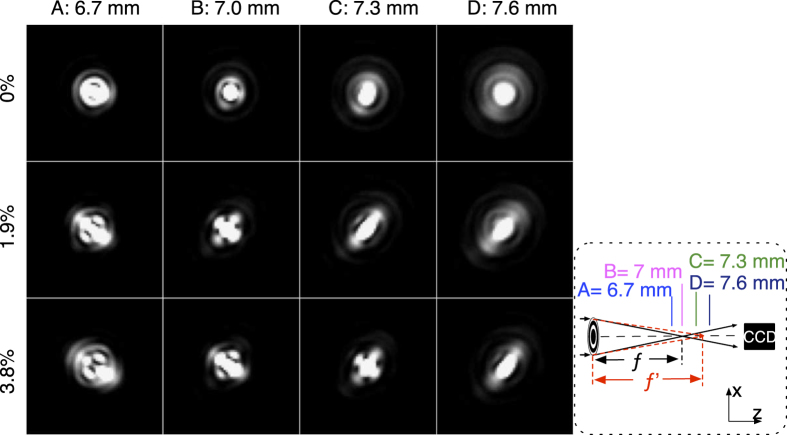
Diffraction patterns captured with a CCD camera at four different positions along the z-axis, demonstrating the effect of stretching the lens. Bright light spot appears at 7 *mm* in the relaxed state of the lens; When we apply a small strain of 1.9% on the lens we observe changes in the diffraction patterns which can be identified as astigmatism; When the strain increases to 3.8%, we observe a focused light spot shift from 7 *mm* at relaxed state to around 7.3 *mm*. This indicates focus change has been realized due to the stretch. The aforementioned four positions are indicated as **A–D** in the inset figure.

The changing diffraction patterns were measured to demonstrate the focal length changes when stretching the Fresnel lens. As the lens is stretched in radial direction with increments of 1.9%, a theoretical increase of 3.88% of the focal length *f* is expected. Figure 6 shows diffraction patterns captured at four positions along z-axis, indicated by A-D in the inset figure at different amounts of applied strain. The diffraction patterns of the lens at relaxed state are in accordance with Fig. 5, and the bright light spot was found at 7 *mm* in Fig. 6, which shows the initial focus point. After stretching the lens along the radial direction by 1.9%, clear diffraction pattern changes can be found at the same positions, even though the focal point stays at around 7 *mm*. As strain goes to 3.8%, the diffraction patterns experience greater changes, and the focused pattern has shifted from 7 *mm* to around 7.3 *mm*, which indicates the focus point changed due to the stretch. Astigmatism was noticed during stretching. The focus spot with astigmatism can be determined when the two axis of the cross are completely symmetric as described in ref. 22.

**Figure 7:**
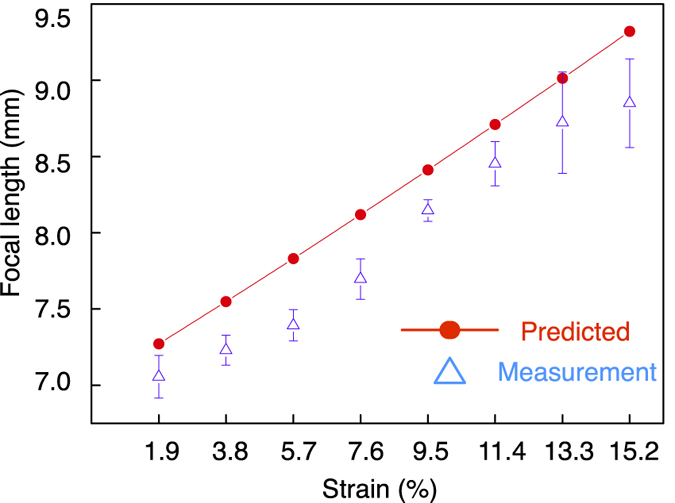
Measurement of the increase in focal length as the lens is stretched radially by the clamps on the holder. The measured focus change follows the predicted focus change. Straining the lens from 1.9% to 11.4%, causes the focal point to shift from 7 *mm* to 8.7 *mm*. The diffraction pattern becomes more distorted when strain is increased further to 13.3% and 15.2%, which is near the maximum extension of the lens. The data are presented as the mean ± standard deviation.

The focus change performance of the lens is shown in Fig. 7, the error bars indicate the standard deviation (SD). The focal length is measured by recording the distance from the lens plate to the focus point, while strain is applied on the sample; increasing from 1.9% to 15.2% with increments of 1.9%. The focal length increases with the strain, which agrees with the computational simulation. For instance, a strain of 11.4%, the focal point shifts from 7 *mm* to 8.7 *mm*, indicating that a change of 24% is achieved. A small SD of around 0.1 *mm* is obtained when the strain ranges from 1.9% to 11.4%. However, when the strain increases to 13.3% and 15.2%, the SD becomes larger than 0.29 *mm*, the diffraction pattern gets distorted and the focal point is less clear. This indicates the limit of the lens with respect to the maximum amount of strain.

## Discussion

Figure 7 shows that a focus change can be achieved as lens size increases due to stretching. The measured focus change follows the same trend as the prediction made by Equation 1. In addition, the results agree with the computed simulation of the focus change before and after stretching (Fig. 2c). This validates the proposed concept for a tuneable Fresnel lens through radial stretching.

**Figure 8:**
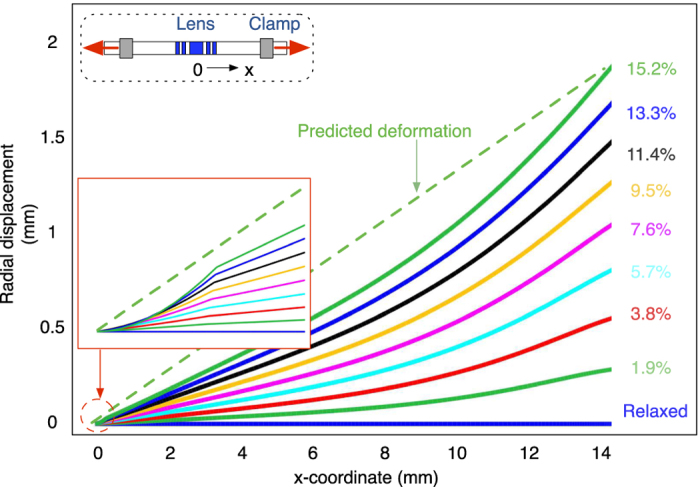
Simulation of the lens deformation in radial direction when the lens is stretched. The non-linear deformation of the polymeric substrate makes the deformation of the lens area near the center smaller than the linear prediction. The close up view (inset) shows that in the center area, a clear difference between the substrate deformation and the predicted value is experienced by the lens.

A mismatch of the focus change between the theoretical calculation and the measurement can be observed in Fig. 7. A possible explanation is the somewhat non-linear deformation of the elastomeric PDMS substrate. During the stretching of the lens, the strain distribution in radial direction is not linear. Simulation of the lens deformation is reported in Fig. 8. The solid lines show the non-linear deformation of the elastomeric PDMS when different strain is exerted, while the dashed line shows a perfect linear distribution of the deformation. For example, as depicted in the close up view, for a strain of 15.2%, the deformation in the lens area is less than the predicted value, resulting in a smaller value of the zone radius for the diffractive lens, hence smaller focus change. This explains why the measured focal change is less than expected.

A SD of 0.14 *mm* is observed at the first stretching for small strains of 1.9% in Fig. 7. Clamping the lens on the holder generates some initial stress near the six clamps which causes some non-uniform deformation of the lens. While stretching the lens, the initial stress of the composite will be either compensated or covered by the stretching, which is unpredictable due to the limited control in the loading of the sample in the holder. This accounts for the large SD for the first stretching. When the stretching continues, the strain from the polymer deformation is dominant and the focal length changes with stretching accordingly. This behaviour is reflected in the data reported in Fig. 7 where a clear focal change is shown when strain goes from 3.8% to 11.4%, and with low SD of around 0.1 *mm*.

Astigmatism is observed during the stretching, as shown by the elliptic patterns in Fig. 6. This is due to the somewhat non-uniform clamping of the lens in the sample holder. The diffractive patterns are very sensitive to the uniformity of the Fresnel lens. The SD of our measurement is increasing for larger applied strains because imperfections in uniformity are increased for larger strains. Secondly, the sample is unloaded, and reloaded after each experiment so the sample can relax back towards its original configuration without being clamped. We assume that the re-positioning of the lens, in combination with the somewhat non-uniform clamping contributes to the SD to the data. We recommend to integrate an actuator which can provide a uniform radial deformation of the lens (similar to a diaphragm), which doesn’t require mechanical clamps.

The proposed Fresnel lens formed by embedding vertically aligned CNT bundles in a PDMS layer, utilizes the optical absorption properties of the CNT and the transparency and flexibility of the PDMS. Diffraction patterns of the lens were measured by a CCD camera to characterize the Fresnel lens. By stretching the lens radially, clear diffraction pattern changes were observed, which were in accordance with the simulation results. A maximum focus change of about 24% can be realized by stretching the lens radially 11.4%. Some imperfections of the clamping mechanism employed to stretch the lens introduced astigmatism. However, we think this problem can be solved by integrating an circular in-plane actuator, which can provide a more uniform deformation of the lens. The focus change obtained from the stretching agrees with the theoretical calculation, making it possible to predict the focus changing performance, which can assist in the design of stretchable Fresnel lenses. We demonstrate a straightforward approach for microfabrication of a tuneable Fresnel lens, making it compatible for the miniaturization and suitable for mass fabrication. Furthermore, the good optical performance of the tuneable diffractive lens indicates great potential in holography. Finally, the bio-compatibility of the polymeric composites makes it promising for integration in biological applications in such as electronic eyes.

## Methods

### Imaging setup for diffractive pattern

A 635 *nm* laser was used to illuminate the lens. A collimating setup, including two convex lenses, a filter and a diaphragm, was used to collimate the light. The Fresnel lens was clamped on a customized holder, which stretched the lens in radial direction. Six symmetrically distributed clamps are maintaining the sample with lens array on the holder. Each clamp can be moved by increments of 0.289 *mm* in radial direction. Considering the full sample is 30 *mm* in diameter, the radius of the lens pattern will increase by 1.9% with each increment. Diffraction patterns were captured by a CCD camera with two additional convex lenses after the light propagates through the Fresnel lens.

### Diffractive pattern analysis

50 shots of diffractive patterns from z = 5.6 *mm* to 8.05 *mm* are captured with a CCD camera, with a step-size of Δz = 0.05 *mm* for each image. The diffraction patterns are normalized (white represents the maximum light intensity and black is the off state of the camera). The intensity profiles from the x-axis (y = 0) of each image are then extracted and reconstructed to an intensity map with Matlab R2015a (Mathworks, B.V.), as shown in Fig. 5g. The focus position with astigmatism can be determined when the two axis of the cross are completely symmetric^22^. The focal length of the lens is measured by recording the distance from the lens plate to the focal point.

### Statistical analysis

The focus change measurement under stretching was repeated on the same lens for three times. The focal point changes of the lens were recorded and analysed. Data are expressed as the mean ± standard deviation, as indicated in corresponding Fig. 7.

### Simulation of the lens deformation

Finite element analysis software (COMSOL Multiphysics 5.1) was used to analyse the deformation of the polymeric substrate. Solid Mechanics model under Structural Mechanics is used for the calculation. The material of the lens model was set to be PDMS. The effect of the CNT are not included because the thickness of PDMS (2 *mm*) is much higher than the height of CNT (10 *μm*). A hyper-elastic material model was used to accurately describe the non-linear material behaviour under moderate strains. The radial symmetric lens model has a radius of 15 *mm* and a thickness of 2 *mm*. A symmetry boundary constraint was added to the centre of the lens, while the prescribed displacement was enforced on the peripheral boundary. The displacement range increased from 0 to 2.32 *mm* with a step size of 0.289 *mm*.

## Additional Information

**How to cite this article**: Li, X. *et al.* Stretchable Binary Fresnel Lens for Focus Tuning. *Sci. Rep.* **6**, 25348; doi: 10.1038/srep25348 (2016).
